# Characterization of synergistic anti-cancer effects of docosahexaenoic acid and curcumin on DMBA-induced mammary tumorigenesis in mice

**DOI:** 10.1186/1471-2407-13-418

**Published:** 2013-09-13

**Authors:** Rafat A Siddiqui, Kevin A Harvey, Candace Walker, Jeffrey Altenburg, Zhidong Xu, Colin Terry, Ignacio Camarillo, Yava Jones-Hall, Cary Mariash

**Affiliations:** 1 Cellular Biochemistry Laboratory, Indiana University Health, Indianapolis, IN 46202, USA; 2 Methodist Research Institute, Indiana University Health, Indianapolis, IN 46202, USA; 3 Department of Medicine, Indiana University School of Medicine, Indianapolis, IN 46202, USA; 4 Department of Biology, Purdue University, West Lafayette, IN 47907, USA; 5 Department of Comparative Pathobiology, Purdue University, West Lafayette, IN 47907, USA

**Keywords:** *In vivo* studies, Cancer cell differentiation, Breast cancer, Tumor incidence, Tumor growth, Maspin, Survivin

## Abstract

**Background:**

The major obstacles to the successful use of individual nutritional compounds as preventive or therapeutic agents are their efficacy and bioavailability. One approach to overcoming this problem is to use combinations of nutrients to induce synergistic effects. The objective of this research was to investigate the synergistic effects of two dietary components: docosahexaenoic acid (DHA), an omega-3 fatty acid present in cold-water fish, and curcumin (CCM), an herbal nutrient present in turmeric, in an *in vivo* model of DMBA-induced mammary tumorigenesis in mice.

**Methods:**

We used the carcinogen DMBA to induce breast tumors in SENCAR mice on control, CCM, DHA, or DHA + CCM diets. Appearance and tumor progression were monitored daily. The tumors were harvested 15 days following their first appearance for morphological and immunohistological analysis. Western analysis was performed to determine expression of maspin and survivin in the tumor tissues. Characterization of tumor growth was analyzed using appropriate statistical methods. Otherwise all other results are reported as mean ± SD and analyzed with one-way ANOVA and Tukey’s post hoc procedure.

**Results:**

Analysis of gene microarray data indicates that combined treatment with DHA + CCM altered the profile of “PAM50” genes in the SK-BR-3 cell line from an ER^-^/Her-2^+^ to that resembling a “normal-like” phenotype. The *in vivo* studies demonstrated that DHA + CCM treatment reduced the incidence of breast tumors, delayed tumor initiation, and reduced progression of tumor growth. Dietary treatment had no effect on breast size development, but tumors from mice on a control diet (untreated) were less differentiated than tumors from mice fed CCM or DHA + CCM diets. The synergistic effects also led to increased expression of the pro-apoptotic protein, maspin, but reduced expression of the anti-apoptotic protein, survivin.

**Conclusions:**

The SK-BR-3 cells and DMBA-induced tumors, both with an ER^-^ and Her-2^+^ phenotype, were affected by the synergistic interaction of DHA and CCM. This suggests that the specific breast cancer phenotype is an important factor for predicting efficacy of these nutraceuticals. The combination of DHA and CCM is potentially a dietary supplemental treatment for some breast cancers, likely dependent upon the molecular phenotype of the cancer.

## Background

The idea that dietary changes or diet supplementation may improve the health of cancer patients or enhance the effectiveness of existing treatments is compelling motivation for exploring the activities of dietary compounds. Although natural products are a promising addition to current toxic anti-cancer drugs, major obstacles exist to the successful use of individual nutritional compounds as preventive or therapeutic agents: efficacy and bioavailability. One approach to overcoming these problems is to use combinations of nutrients with synergistic effects. Given that the human diet consists of multiple nutrients, it is likely that nutrients in the diet act synergistically to provide health benefits. In fact, human diets can routinely encompass many biologically active small molecules, and evidence for synergy between dietary compounds is emerging [1-3]. The translational benefit for such molecules derives from a relative lack of toxic side effects and source material that is inexpensive and easily accessible relative to synthetic pharmaceuticals. The objective of the present research is to establish synergistic interaction with a combination of Docosahexaenoic acid (DHA), an omega-3 PUFA found in fish oil, and curcumin (CCM), a phenolic molecule found in turmeric, on breast cancer growth.

Docosahexaenoic acid (22:6^Δ4,7,10,13,16,19^) is the most unsaturated of the fatty acids commonly found in biological systems. Early epidemiological evidence strongly links fish oil (rich in DHA and eicosapentaenoic acid [EPA]) with a low incidence of several types of cancer, including breast cancer [4-7]. In addition to strong epidemiological studies, dietary studies have also substantiated DHA’s role as an anti-cancer agent for breast cancer [8-10]. Curcumin [1,7-bis(4-hydroxy-3-methoxy phenyl) -1,6-heptadiene-3,5-dione] has been frequently used in South Asian medicine since the second millennium BCE. Coincidently, a recent study reported that breast cancer rates in India were significantly lower than in Western countries, including the US [11]. Preclinical studies have revealed growth-inhibitory potential of curcumin in several cancers, including colon, duodenal, stomach, prostate, and breast [8,12-17].

Breast cancer is a myriad of diseases with multiple phenotypes. Clinically, breast cancers are subdivided according to estrogen receptor (ER) and oncogenic Her-2 status. Progesterone receptor (PR) is another molecular marker that is also used to predict a lack of response to hormone therapy [18]. More recent studies using global gene expression profiling with widely available microarray techniques describe distinct molecular subtypes of breast cancer, each defined by a large number of genes [19-21]. These include basal-like, Her2-enriched, normal-like, luminal A, and luminal B subtypes. This classification has been further refined and now utilizes a set of 50 representative genes known as “PAM50” genes [22,23]. Those classifications also parallel the established clinical- and histological-based classifications, with basal-like representing ER^-^/Her2^-^cancers, Her-2 enriched representing ER^-^/Her2^+^, and normal-like and luminal A/B subtypes representing ER^+^. With this diverse classification, it would be expected that a particular therapeutic agent or dietary supplement might not be effective for all malignant subtypes. Although there is a debate about the advantage of molecular signature classification over existing surface receptor classification [24-26], the molecular signature may provide more in-depth knowledge about the progression of disease or response to treatment.

In a previous study, we used 5 breast cell lines covering distinct receptor expression phenotypes: MDA-MB-231 (ER^-^ PR^-^ Her2^-^), SK-BR-3 (ER^-^ PR^-^ Her2^+^), MCF7 (ER^+^ PR^+^ Her2^-^), MDA-MB-361 (ER^+^ PR^-^ Her2^+^), and MCF10AT (ER^+^, PR isoform B but not A, Her2 variable) [27-30]. Across these cell lines, the synergistic anti-proliferative effects of CCM, DHA, and a DHA + CCM combination were assessed quantitatively as described by Tallarida [31]. Our data demonstrated that the combination of DHA + CCM (3:2), when less than 50 μM, exerted a synergistic effect only in the SK-BR-3 breast cancer cell line. Detection of anti-proliferation synergy for DHA + CCM within the SK-BR-3 cell line was followed by transcript analysis using the Agilent Whole Human Genome Microarray 4×44K platform. The microarray data and corresponding step-by-step analysis is posted as supplementary data on the BMC-Cancer web site [32]. The data demonstrate that the expression of genes involved in apoptosis, inhibition of metastasis, and cell adhesion were upregulated, whereas genes involved in cancer development and progression, metastasis, and cell cycle progression were downregulated [32]. Furthermore, a significant 20- to 100-fold increase in *CYP450 class-1,* a nearly 20-fold upregulation of *SERPINB5*, and a 60% downregulation of *BIRC5* gene expression are of special functional interest. CYP450 proteins are involved in the metabolism of estrogen, activation/inactivation of carcinogens, and enhancement of the anti-proliferative effects of polyphenols [33-39]. SERPINB5 protein (also known as maspin, mammary gland-associated serine protease inhibitor) is a pro-apoptotic tumor suppressor that is completely suppressed in most breast cancers but is re-expressed on anti-cancer treatment [40], whereas the BIRC5 protein (also known as survivin), belongs to the Inhibitors of Apoptosis Protein (IAP) family, which is mostly absent from well-differentiated, normal adult tissues, but is over-expressed in nearly all human cancers [41]. The fact that only the SK-BR-3 cell line was synergistically affected by DHA and CCM suggests that specific breast cancer phenotype is an important factor for predicting efficacy.

We used the microarray data to further analyze and understand the response of dietary treatments on “PAM50” genes. We made initial attempts to test the synergism between DHA and CCM in a xenograft model of the SK-BR-3 cell line; however, we were not able to grow the SK-BR-3 xenograft in nude mice because of low tumorigenic potential of SK-BR-3 cells. Therefore, in the present study we present results from an *in vivo* study on DMBA-induced ER-negative/Her-2 positive breast tumors to validate the DHA and CCM synergistic effects in a similar phenotypic breast cancer.

## Methods

### Materials

SK-BR-3 cells were obtained from the American Type Culture Collections (ATCC; Manassas, VA) and maintained in McCoy’s 5A medium (ATCC) supplemented with penicillin (100 units/ml), streptomycin (100 μg/ml), and 10% FBS. McCoy’s 5A medium, penicillin, streptomycin, and glutamine were from Invitrogen Corporation (Grand Island, NY). Fetal bovine serum was from BioWhittaker (Walkersville, MD). DHA (NuChek Prep, Inc., Elysian, MN) was diluted in 100% ethanol to make 50 mM stock solutions. CCM (Sigma Aldrich, St. Louis, MO) was dissolved in DMSO to make 50 mM stock solutions. The fatty acid standards for gas chromatography (GC) were from Nu-Chek Prep, Inc. (Elysian, MN). Docosahexaenoic acid single cell oil (DHASO) was a generous gift from DSM Nutrition (Columbia, MD). Methanol, chloroform, petroleum ether, diethyl ether, acetic acid, hexane, and ethanol were from Fisher Scientific (Fair Lane, NY). Anti mouse ER, Her-2 and PR antibodies were from Santa Cruz Biotechnology Inc. (Dallas, TX). H & E stain and all other reagents were from Sigma Chemical Co. (St Louis, MO).

### Animals and diets

One week after receiving the animals, SENCAR (SENsitive to CARcinogen) mice (female, 3 weeks old, 25-30 g, Frederick National Laboratory for Cancer Research, National Cancer Institute, Fredrick, MD) were randomly divided into 4 groups and fed *ad libitum* diets containing corn oil (control diet), corn oil with CCM (CCM-diet), DHASCO (DHA-diet), or DHASCO with CCM (DHA + CCM-diet) (Taklad, Harlan laboratories, Madison, WI, USA) for 3 weeks prior to tumor induction. Mice continued feeding on the corresponding diets and were weighed every week throughout the study. The diets contained similar quantities of protein (20% of calories), carbohydrates (42% of calories), lipids (38% of calories), vitamins, and minerals as described in Table 1. They only differed in the types of lipids (i.e., corn and DHASCO) and their fatty acids composition as described in Table 2. At six weeks of age, the mice were gavaged with 200 μl of DMBA (1 mg/ml in sesame oil) one time per week for six weeks [42,43]. Mice were examined daily for the appearance of tumor by palpation, and the first day of tumor detection was recorded. Mice were anesthetized using Isoflurane 15 days after the first appearance of tumor. A blood specimen was collected by cardiac puncture, and the tumor was dissected out, measured, and weighed. Blood and tumor specimens were stored at −70°C. A portion of the tumor tissues was embedded in OCT (optimal cutting temperature) compound for immunohistology for ER, PR, and Her-2 expression and histological evaluation by hematoxylin and eosin (H&E) stain. The protocol for these studies was approved (protocol # 2010–22) by the Methodist Research Institute’s Animal Research Committee (Animal Welfare Assurance Number-A3772-010) and strictly followed *Guide for the care and use of laboratory animals* (NIH publication No.85-23, revised 1996)*.*

**Table 1:** Formulation of experimental diets

	**Corn oil**	**Corn oil + CCM**	**DHASCO**	**DHASCO + CCM**
	**g/Kg**			
**Casein**	235	235	235	235
**DL-Methionine**	3.5	3.5	3.5	3.5
**Corn Starch**	247	245	247	245
**Maltodextrin**	130	130	130	130
**Dextrose**	90	90	90	90
**Corn oil**	180	180	30	30
**DHASCO**	0	0	150	150
**CCM**	0	2	0	2
**Celluloase**	59	59	59	59
**Mineral Mix**	41	41	41	41
**Vitamin Mix**	12	12	12	12
**Choline Bitartrate**	2.4	2.4	2.4	2.4
**Vitamin E (1100 IU/g)**	0.0075	0.0075	0.0075	0.0075
**Vitamin C (35%)**	0.05	0.05	0.05	0.05

**Table 2:** Fatty acid composition of the experimental diets

	**Corn oil**	**Corn oil + CCM**	**DHASCO**	**DHASCO + CCM**
**C14:0**	0.13 ± 0.00	0.14 ± 0.00	9.83 ± 0.15	9.62 ± 0.13
**C16:0**	11.75 ± 0.23	11.75 ± 0.30	10.39 ± 0.14	10.42 ± 0.05
**C16:1n-7**	0.10 ± 0.00	0.10 ± 0.00	1.88 ± 0.01	1.82 ± 0.02
**C18:0**	1.70 ± 0.09	1.69 ± 0.02	1.01 ± 0.01	1.34 ± 0.26
**C18:1n-9**	27.06 ± 0.50	27.03 ± 0.75	23.85 ± 0.31	23.38 ± 0.23
**C18:2n-6**	56.81 ± 1.04	56.83 ± 1.70	11.11 ± 0.15	10.99 ± 0.16
**C18:3n-3**	0.94 ± 0.02	0.94 ± 0.03	0.20 ± 0.00	0.20 ± 0.00
**C20:0**	0.35 ± 0.01	0.35 ± 0.01	0.15 ± 0.01	0.14 ± 0.01
**C20:1n-9**	0.24 ± 0.01	0.25 ± 0.01	0.10 ± 0.00	0.10 ± 0.00
**C20:5n-3**	0.06 ± 0.00	0.06 ± 0.00	0.14 ± 0.01	0.13 ± 0.01
**C22:6n-3**	0.04 ± 0.00	0.03 ± 0.01	34.81 ± 0.73	35.44 ± 0.46
**Total-SFA**	14.02 ± 0.32	14.03 ± 0.33	26.97 ± 0.41	26.99 ± 0.55
**Total MUFA**	27.64 ± 0.53	27.63 ± 0.78	24.40 ± 0.38	23.95 ± 0.26
**Total n-6 PUFA**	56.81 ± 1.00	56.83 ± 1.70	11.11 ± 0.15	10.99 ± 0.16
**Total n-3 PUFA**	1.05 ± 0.03	1.034 ± 0.04	35.52 ± 0.75	36.15 ± 0.49
**n-6/n-3**	54	55	0.31	0.30

### Whole breast mount

The entire intact lower abdominal mammary gland (#4) was dissected out and spread on a glass slide for measuring the size and histological evaluation as described [44]. The gland was air dried briefly and then fixed in Carnoy’s fixative (6 parts 100% ethanol, 3 parts methanol and 1 part glacial acetic acid) overnight. The mount was rehydrated in increasing dilutions of ethanol in distilled water (70%, 50%, 30%, 10%, 0%, 10 minutes each) and then stained by placing the slide in Carmine Alum stain over night. The excess stain was removed by washing with increasing concentrations of ethanol (70%, 95%, 100%, 15 minutes each), and then the slides were placed in xylene solutions for at least 2 days until the fats were sufficiently cleared from the gland. The mammary tissue was mounted using Fluoromount and a glass cover slip. Images were recorded using a dissecting microscope (Leica S8APO, Leica Corporation, Switzerland), and photographs were captured with a digital camera (MagnaFire, Optronics, Goleta, CA).

### Histology

Transverse serial sections of tumor tissues (10 μm) were prepared using a cryostat (Leica CM1900, Leica Microsystems, Bannockburn, IL). The analysis of tissue histology was performed by staining sections with H & E stain (Sigma Chemical Co., St Louis, MO). Slides were examined by Dr. Yava Jones in the Department of Comparative Pathobiology at Purdue University. The tumors were classified based on their morphological features as described by Dunn [45]. For detecting ER, PR, and Her-2 expression, immunohistology was performed by the pathological laboratory services of Indiana University Health (Indianapolis, IN) using mouse specific anti-estrogen receptor, progesterone receptor, and Her-2 receptor antibodies. Slides were scanned and the expression of ER, PR, and Her-2 was quantified using Aperio ImageScope software (Aperio, Vista, CA). The positive stained area and total scanned area were measured with precise calibration, and the percent of the positive stained area was determined. The total scanned area excludes the uneven tissue edges and void regions without cells. Expressions of antigens in CCM, DHA, and DHA + CCM are reported as fold changes compared to control (corn oil fed animals).

### Western blot analysis

The tumor tissues were homogenized in a homogenizing buffer (0.25 M sucrose, 50 mM Hepes, pH 7.4, 2 mM EGTA) using a polytron homogenizer. The homogenate was solubilized in 2× lysis buffer (20 mM Tris–HCl, pH 7.4, 137 mM NaCl, 100 mM NaF, 2 mM Na_3_VO_4_, 10% glycerol, 1% nonidet P-40, 2 mM PMSF, 1 μg/mL leupeptin, 0.15 units/mL aprotinin and 2.5 mM diisofluorophosphate) for 10 minutes on ice. The detergent solubilized extracts were centrifuged to remove insoluble matter. After evaluating the protein content using a BCA (bicinchoninic acid) Protein Assay Kit (Pierce, Rockford, IL), 15 μg of protein solubilized in Laemmli sample-loading buffer was loaded onto each lane of a 4-12% gradient SDS-polyacrylamide gel and transferred onto nitrocellulose membranes. Membranes were blocked for 30 minutes at room temperature in 10% Roche western blocking reagent in Tris buffered saline supplemented with 0.1% Triton X-100 (TBST). Blots were probed with primary antibodies (anti-maspin, anti-survivin, Cell Signaling Technology, Danvers, MA; anti-β-actin, Santa Cruz Biotechnology, Dallas, TX) according to the manufacturer’s recommendations. Secondary antibodies were peroxidase-conjugated for protein detection using an enhanced chemiluminescence (ECL) system (Amersham Pharmacia Biotechnology, Piscataway, NJ, USA). Nitrocellulose membranes were stripped in 62.5 mM Tris–HCl (pH 6.8) buffer containing 2% SDS and 100 mM β-mercaptoethanol for 30 minutes at 50°C. Stripped blots were washed 6 times in TBST, blocked, and reprobed with an alternative antibody.

### Statistical analysis

Data is presented as mean ± SD unless reported otherwise. The progression of tumor development in different dietary groups was compared using the Chi-square test, whereas the number of tumors formed/animal in each group was compared between groups using one-way ANOVA with Scheffe post hoc test. Data for time to initial tumor appearance are summarized as median (Q1, Q3) and compared between groups using log-rank test. All other comparisons were made by one-way ANOVA with Tukey’s post hoc test using IBM SPSS statistics 20 software.

## Results

### Effect of DHA and CCM on “PAM50” gene expression

**Figure 1:**
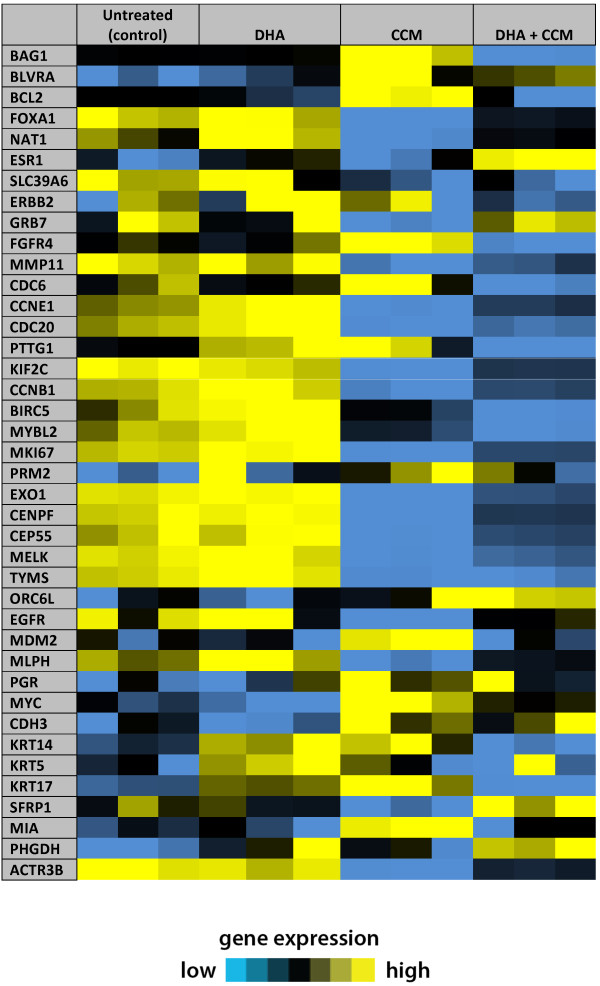
**The signature profile of PAM50 gene expression in SK-BR-3 cells.** The expression profile of PAM50 genes (40 matching genes) from microarray data [32] in SK-BR-3 cells treated with vehicle, DHA, CCM or DHA + CCM were used to compare the signature profile of 41 genes represented on the U133A array system, as reported by Creighton [46], to classify tumors into basal-like, Her-2-enriched, luminal A, luminal B, and “normal-like.” The expression profile of untreated cells (control) resembled the ER^-^, Her-2-enriched profile, whereas the expression profile of cells treated with DHA + CCM more closely resembled the “normal-like” profile.

We used the microarray data from the SK-BR-3 cell line to examine the signature profile of “PAM50” genes and determine if the combined treatment with DHA and CCM influenced the expression of the gene signature profile. The data presented by Creighton [46] and Hoadley [47] represents a modified gene signature profile for breast cancer sub-classification. We selected the same genes from our microarray data (Figure 1) and arranged them in a similar manner, as described by Creighton [46]. We found that the gene signature of SK-BR-3 cell lines resembled the ER^-^/Her-2^+^ tumor profile, further confirming our SK-BR-3 cell characterization. DHA alone had very little effect, but CCM treatment changed the expression of a number of genes. DHA, however, appears to be acting as a modulator of the effects of CCM, and it is very intriguing to observe that the combined DHA + CCM treatment has altered the SK-BR-3 profile from an ER^-^/Her-2^+^ (untreated cell) phenotype to resemble a “normal-like” phenotype. Furthermore, as shown in Table 3, DHA or CCM alone has no significant effect on ER, Her-2, and PR expression; however, the DHA and CCM combination caused a nearly 3-fold increase (*P* < 0.001) in ER expression, whereas DHA or CCM alone had no effect. This observation was further validated in our *in vivo* experiments presented below.

**Table 3:** Changes in estrogen receptor, progesterone receptor and her-2 oncogenes in SK-BR-3 cell and DMBA-induced tumors

	**DHA + CCM**	***P *value**	**CCM**	***P value***	**DHA**	***P *value**
**Gene expression (SK-BR-3 cells)**						
	**(n = 3)**		**(n = 3)**		**(n = 3)**	
***PGR***	1.67	0.628	2.05	0.294	1.16	0.991
***ESR-1***	2.90	0.00 1	1.04	0.994	1.19	0.581
***ERBB2***	0.97	0.119	1.01	0.187	1.08	0.227
**Protein expression (DMBA-induced tumors)**						
	**(n = 4)**		**(n = 10)**		**(n = 10)**	
**PR**	0.38	0.913	0.67	0.962	0.80	0.992
**ER-alpha**	7.50	0.01	3.81	0.219	3.41	0.415
**Her-2**	2.60	0.261	1.68	0.704	0.97	1.00

Values are fold changes compared to vehicle treated control. The data is analyzed by one-way ANOVA and Tukey’s post hoc test.

### Effects of Curcumin and DHA on tumor development

**Figure 2:**
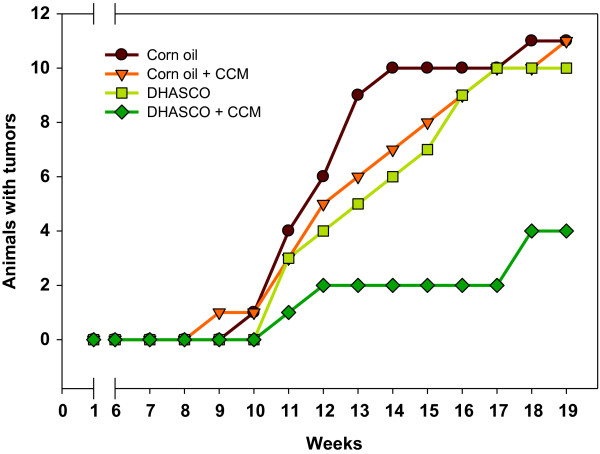
**Effect of DHA, CCM, and DHA + CCM on DMBA-induced breast tumor development.** After an acclimation period, SENCAR mice (NIH, Fredric, MD) were divided into 4 groups (15/group). Each group was fed a different diet for 3 weeks prior to tumor induction: 1) 18% corn oil (Brown line); 2) 15% DHASCO (DSM, Columbia, MD) + 3% corn oil (Light green line); 3) 18% corn oil + 0.2% curcumin (Orange line); or 4) 15% DHASCO + 3% corn oil + 0.2% curcumin (Dark green line). Doses of DHA and CCM were selected based on published data [2]. Mice continued to be fed the corresponding diet during the entire course of the experiment. Each mouse was gavaged with 200 μl DMBA (1 mg/ml in sesame oil) once every week for 6 weeks to induce breast tumors. The appearance of palpable tumors was monitored daily beginning with the first DMBA gavage. The statistical analysis and characterization of the effects of different diets on DMBA-induced breast tumors are shown in Table 4.

**Figure 3:**
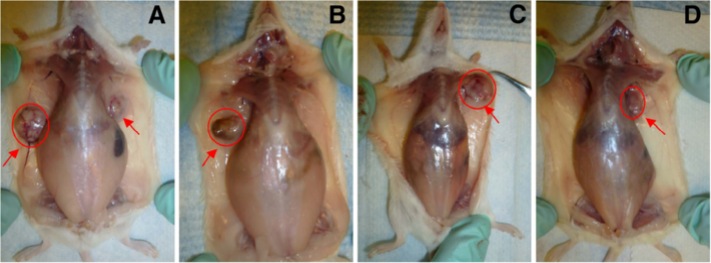
**Size and location of DMBA-induced tumors in different dietary groups.** The details of animals and tumor induction are given in the legend of Figure 2. Sites of tumor development in animals fed a corn oil-diet **(A)**, CCM-diet **(B)**, DHA-diet **(C)** or DHA + CCM-diet **(D)** are shown by red arrows. Red circles indicate relative tumor sizes.

The data presented in Figure 2 demonstrate that a DHA or CCM diet alone did not reduce the incidence of tumor occurrence in mice, whereas the combined DHA diet with CCM significantly delayed tumor initiation and also significantly reduced the incidence of breast tumor in mice. The data presented in Table 4 indicate that about 73% of mice on the corn oil and corn oil + CCM diets developed tumors, and mice on the DHA diet yielded a tumor incidence of 67%. However, only 27% (*P* = 0.0240) of animals developed tumors when on the DHA + CCM diet. There was no statistical difference in the number of tumors per animal within corn oil, CCM, and DHA groups; however, there were significantly fewer breast tumors per animal when treatment with DHA and CCM was combined. In addition, the average tumor mass (Table 4 & Figure 3) in the DHA + CCM group was also significantly less (0.3 g) compared to other groups (1.2 - 1.4 g) (*P* = 0.026). Furthermore, the length of time for the initial tumor to appear in animals fed DHA + CCM was significantly longer (*P* = 0.018) than that of animals fed control, DHA, or CCM diets. The DHA, CCM, or DHA + CCM treatment was non-toxic, based on the lack of significant differences in body weights between groups (data not shown).

**Table 4:** Characteristics of DMBA-induced tumors in SENCAR mice on different dietary treatment

	**Corn oil**	**Corn oil + CCM**	**DHASCO**	**DHASCO + CCM**	***P *value**
**Number of mice developing tumors**^**&**^	11 (73.3%)	11 (73.3%)	10 (66.7%)	4 (26.7%)	0.024
	(n = 15)	(n = 15)	(n = 15)	(n = 15)	
**Number of tumors/animal**^**#**^	1.27 ± 1.03	1.20 ± 1.01	0.87 ± 0.74	0.27 ± 0.45	0.036
	(n-15)	(n = 15)	(n = 15)	(n = 15)	
**Tumor weight**^**+ **^**(g)**	1.1 ± 0.6	1.3 ± 0.8	1.4 ± 0.3	0.3 ± 0.3	0.026
	(n = 11)	(n = 11)	(n = 10)	(n = 4)	
Time to tumor development^* ^**(days)**	80 (70, 87)	87 (73, 108)	92 (77, 101)	NE (114, NE)	0.018
	(n = 15)	(n = 15)	(n = 15)	(n = 15)	

& compared between groups using Chi-square test;

+ compared between groups using ANOVA;

# compared between groups using one-way ANOVA with Scheffe post hoc test.

* Data summarized median (Q1, Q3) and compared between groups using the log-rank test.

*NE* not estimable.

### Effects of curcumin and DHA on breast development

**Figure 4:**
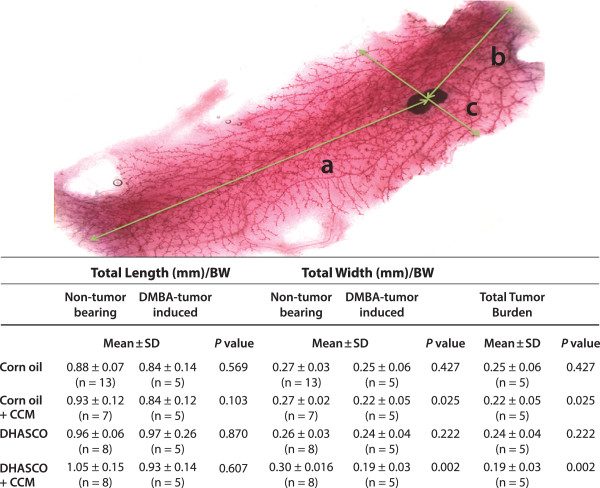
**Effect of diets on DMBA-induced tumors in SENCAR mice.** The total length (a+b) and width (c) were measured as indicated in the total breast mount picture. The total length and width were compared between non-tumor-bearing and DMB-induced tumor groups, whereas as total tumor burden was calculated by adding the number of palpable tumors (Table 4) and number of proliferative regions (Figure 5) in each animal within a dietary group. Data is analyzed by oneway ANOVA and Tukey post-hoc test.

**Figure 5:**
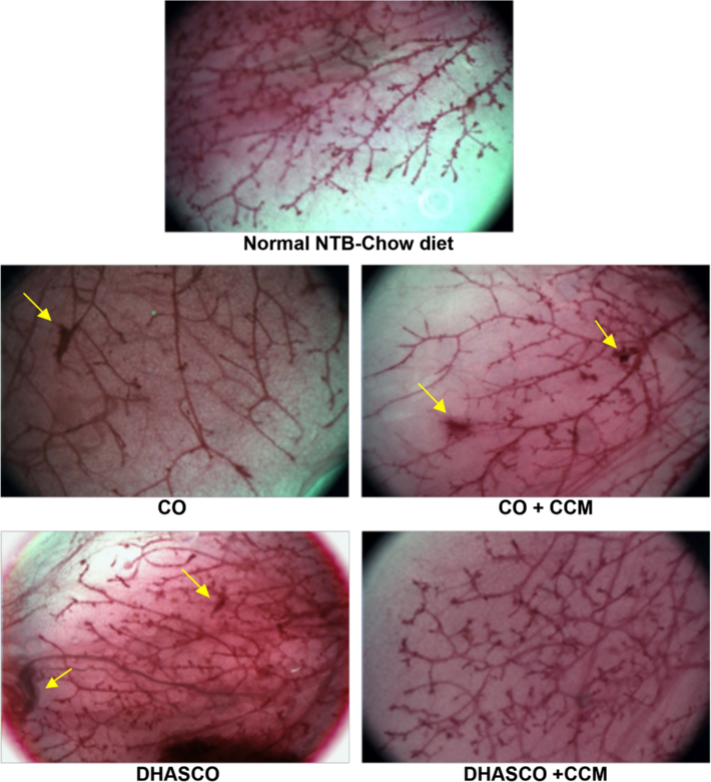
**Effect of diet on the breast tissues morphology.** The details of animals and tumor induction are given in the legend of Figure 2. Breast tissues were isolated from the abdominal region on day 15 after the first appearance of the tumor. Breast tissues from non-tumor-bearing (NTB) mice with a similar age group were used for comparison. The whole breast mounting was performed as described in the experimental section. The tissues were observed under a dissecting microscope (Leica S8APO) at 20× magnification and the hyper-proliferative regions (arrows) in the entire breast tissue were recorded.

We further investigated if the carcinogen or diet had any influence on normal mouse breast development by preparing breast whole mounts (Figure 4). The total length of breast tissue per gram body weight did not differ significantly among the dietary groups with or without DMBA-induced tumors. The total width of breast tissue per gram body weight was not significantly different within DMBA-induced or non-DMBA treated animals. However, the total width of breast tissue per gram body weight was significantly reduced in animals with CCM (P = 0.025) or DHA + CCM (P = 0.002) treatment only in the DMBA-tumor group, whereas the total width of breast tissue per gram body weight was not different on these treatments in non-DMBA induced animals. We also looked at the morphological features of the whole breast mount from animals on different dietary groups (Figure 5). The mammary ducts in control animals (corn oil fed) with DMBA-induced tumors exhibited less differentiation of the gland with substantial reduction in the density of terminal end buds (TEB) compared to animals fed the other diets. Animals on CCM or DHA diets also had some reduction in TEB density compared to control non-tumor-bearing animals, whereas animals on DHA + CCM diets had well differentiated breast tissues and the TEB density was similar to that of control, non-DMBA-induced animals. In addition, we also looked for the presence of proliferative regions where the alveolar buds showed extensive staining. Data presented in Figure 5 show that control animals had an average of 1.3 proliferative lesions per breast. DHA treatment did not affect the number of the proliferative lesions. Although non-significant, the CCM diet reduced proliferative lesions to 40% (0.5 average proliferative lesions/breast), and a DHA + CCM diet substantially reduced proliferative lesions to 20% (0.25 average proliferative lesions/breast) (data not shown). However, the total tumor burden, estimated by adding the palpable tumor and proliferative lesion in each breast tissue (Figure 4) showed a significant 50% reduction (*P* = 0.028) in breast tissue from animals fed a DHA + CCM diet compared to the control group.

### Histology of breast tumors

**Figure 6:**
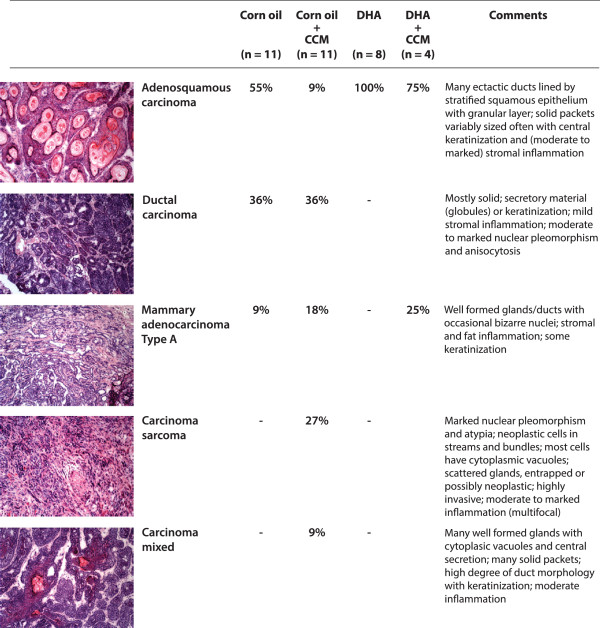
**Histological characterization of DMBA-induced tumors.** The breast tumors were isolated on day 15 after the first appearance of tumor and embedded in OCT. Transverse serial sections of tumor tissues (10 μm) were prepared using a cryostat (Leica CM1900, Leica Microsystems, Bannockburn, IL) and the sections were subjected to H & E stain (Sigma Chemical Co., St Louis, MO). The tumors were classified based on their morphological features as described by Dunn [45].

The basic morphological features of tumors were evaluated using H&E stain. The data presented in Figure 6 indicate that control animals on a corn oil diet largely developed adenosquamous (55%) and ductal (36%) carcinomas, with few tumors showing adenocarcinoma type A (9%). Animals fed a curcumin diet developed mostly ductal carcinoma (36%) and carcinosarcoma (27%), with some tumor showing features of adenocarcinoma type A (18%), whereas only few tumors were adenosquamous carcinoma or mixed carcinoma type (9%). Interestingly, animals fed either a DHA or DHA + CCM diet mostly formed adenosquamous type carcinoma (75%-100%) with marked central keratinization.

**Figure 7:**
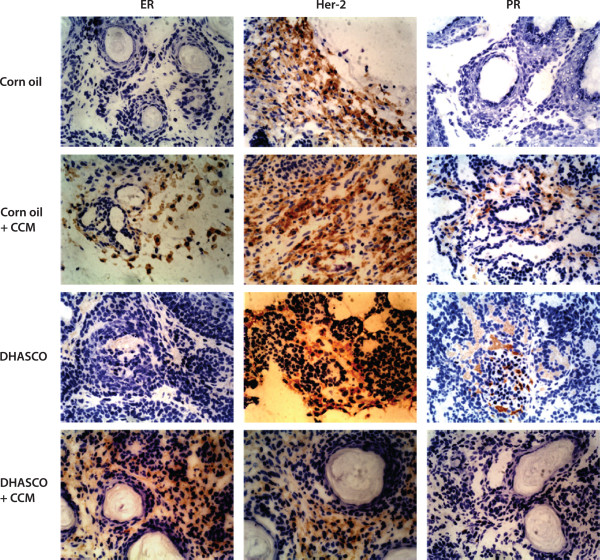
**Effect of diets on the estrogen receptor, Her-2 oncogene, and progesterone receptor expression in DMBA-induced tumors.** Tumors were isolated and tissue sections were prepared as described in the legend of Figure 6. Expression of estrogen receptor (ER), Her-2 oncogene (Her-2), and progesterone receptor (PR) were detected using specific anti-mouse antibodies. Slides were observed under an Olympus microscope at 50 × magnification. The quantitative analysis antigen expression is described in Table 3.

Histological analysis of the tumors indicates that the DMBA tumors were largely ER^-^, HER-2^+^ and PR^-^ (Figure 7). However, when animals were treated with CCM or DHA + CCM, these tumors changed their behavior and were ER^+^, Her-2^+^ and PR^**-**^/^+^ (Figure 7). The quantitative analysis of ER, Her-2 and PR proteins in immunohistological slides is shown in Table 3, which indicates that DHA + CCM treatment caused a significant 7.5-fold increase (*P* = 0.01) in the expression of ER in tumors, whereas none of the other treatments had any effect on the expression of ER, Her-2, or PR.

### Effect of DHA and CCM on maspin and survivin expression

**Figure 8:**
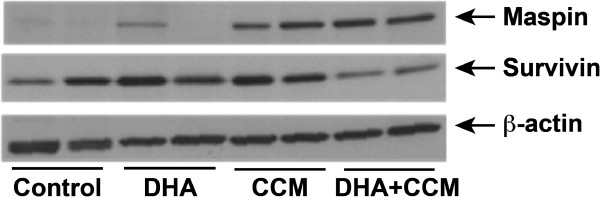
**Effect of diets on expression of maspin and survivin in DMBA-induced tumors.** Frozen tumor tissues were used to extract proteins as described in the text. Expression of maspin and survivin was detected using specific anti-maspin and anti-survivin antibodies. Blots were probed with β-actin antibodies for monitoring sample loading. Blots represent two representative tumors from each group.

As mentioned above, our micro array data indicated a 20-fold increase in *SERPINB5* expression and a 60% reduction in *BIRC5* genes in SK-BR-3 cells treated with DHA + CCM compared to the control cells. We, therefore, analyzed the expression of maspin (*SERPINB5*) and survivin (*BIRC5*) in tumors from different dietary treatments. As demonstrated in Figure 8 using two representative tumors, maspin was absent or expressed at a very low level in a majority of tumors in animals fed either a control (corn oil) or DHA diet; however, a substantial amount of maspin was expressed in tumors from mice fed a CCM diet, and its expression was further stimulated in tumors from DHA + CCM fed animals. In contrast, considerable survivin expression was observed in tumors from animals fed a control diet, a DHA-enriched diet, or a CCM-enriched diet. However, DHA + CCM treatment caused nearly a 50% reduction in survivin expression in the tumors.

## Discussion

About 41% of all newly approved drugs are estimated to have a nutritional/natural product origin, and about 60% of these are anti-cancer drugs [48]. However, it is becoming apparent that the major obstacles to the successful use of individual nutritional compounds as preventive or therapeutic agents are their efficacy and bioavailability. One approach to overcoming this problem is to use combinations of nutrients to induce synergistic effects. Traditionally, nutritional compounds in “folk medicine” are used in unmodified form, as concentrated extracts. Given that the human diet consists of multiple nutrients, dietary nutrients likely act synergistically to provide health benefits. Centuries ago Hippocrates stated, “Let food be thy medicine, and let thy medicine be food." DHA and CCM are natural non-toxic nutrients that have anti-cancer properties; however, their use as individual compounds is not very efficacious. Therefore, we tested the possibility that they could act synergistically. In our previously published *in vitro* studies, we used 5 breast cell lines covering distinct receptor expression phenotypes: MDA-MB-231 (ER^-^ PR^-^ Her2^-^), SK-BR-3 (ER^-^ PR^-^ Her2^+^), MCF7 (ER^+^ PR^+^ Her2^-^), MDA-MB-361 (ER^+^ PR^-^ Her2^+^), and MCF10AT (ER^+^, PR isoform B but not A, Her2 variable). We found that SK-BR-3, an ER^-^/Her-2^+^ cell line, responded synergistically to the DHA + CCM combined treatment [32]. We further demonstrated that the synergistic effects of DHA and CCM were mediated through the activation of NFκB and the expression of PPARγ. As outlined in the introduction, our gene microarray data showed that expression of genes involved in apoptosis, inhibition of metastasis, and cell adhesion were upregulated, whereas genes involved in cancer development and progression, metastasis, and cell cycle progression were downregulated on the combined DHA + CCM treatment. Those data suggested that this differential gene expression by the combined treatment could be effective in limiting growth of cancerous cells.

In addition, we further analyzed the “PAM50” subset of genes to validate the breast cancer signature profile of SK-BR-3 cell lines and to determine if this signature profile changes in response to the combined DHA + CCM treatment. As expected, the untreated SK-BR-3 cells showed a signature pattern for ER^-^, Her-2^+^ tumors. Importantly, we found that DHA + CCM treatment transformed the PAM50 gene signature profile towards a “normal-like” profile (Figure 1) with significant ER expression. This observation indicates that these compounds act synergistically to transform a highly undifferentiated tumor into a differentiated form. We speculate that this concept of chemically changing the gene profile of tumor into “normal-like” tissue will open new avenues to identify the key target genes that may transform a neoplastic cell into a normal cell. The concept of changing cellular structure and function has been published when a differentiated cell was transformed into a stem cell by introducing 4 key genes [49]. It is possible that a reverse approach may have high potential for the treatment of tumors.

In our previous studies on SK-BR-3 cells, we realized that treating breast cancer cells *in vitro* with a combination of DHA + CCM may reflect a similar response *in vivo.* We, therefore, further extended our studies in an *in vivo* model of breast cancer. We initially used a xenograft model of SK-BR-3 tumors in nude mice. Because of the low tumorigenic potential of SK-BR-3 cells, these studies could not be completed. We, therefore, used a DMBA-inducible breast cancer model to determine the effects of DHA, CCM, and DHA + CCM. Interestingly, the DMBA-induced breast cancer model in SENCAR (sensitive to carcinogenesis) mice has been shown by others [50-53] and validated by us, to exhibit a phenotype (ER^-^, Her-2^+^) similar to that of SK-BR-3 cells [30]_._ Therefore, our *in vivo* model closely resembled our *in vitro* breast cancer cell model.

The data presented in Figure 2 demonstrate that DHA in combination with CCM delays tumor initiation and reduces the incidence of breast tumors in mice. Morphologically, breast tumors in the DHA + CCM group appeared to be more differentiated then control tumors. Additionally, the single treatment with either DHA or CCM did not alter the TEB, which were similar to the non-tumor control. No apparent difference was found in the size (length and width) of normal breast tissue in any dietary group, indicating that diet itself has no effect on the development of breast. In contrast, breast tissue width was significantly reduced in DMBA-induced animals fed a CCM or DHA + CCM diet. This indicates a possible interaction of DMBA with CCM, but it is not clear if this reduction in breast width has any pathological implications.

Both DMBA and CCM are metabolized to their active metabolites by cytochrome P450 (CYP450) class 1 enzymes [54,55]. The expression of these enzymes is directly regulated by the activation of Aryl hydrocarbon receptor (AhR). Both CCM and DMBA bind to AhR to induce expression of CYP40-class-1 enzymes [56,57]. It is, therefore, possible that CCM and DMBA may have interacted at the AhR-CYP450-1 axis and that agonist vs antagonist effects of DMBA and CCM may have some growth inhibitory effects on breast development. The role of CCM and DMBA on AhR activation and the metabolism of CCM and DMBA clearly require further investigation.

Histological examination of the breast tumors allowed us to subclassify them into multiple types. The most common tumor type in control- or CCM-treated animals was ductal carcinoma (36%); however, the tumors that developed on a DHA or DHA + CCM diet appeared to be largely an adenosquamous type with marked central keratinization (75-100%). The expression of keratin is a differentiation marker of epithelial cells and plays an essential role in the malignant behavior of breast tumors [58]. Nearly 80% of breast carcinomas exhibit a loss of the differentiation-associated keratin 8 and 18 have generally been associated with a worse prognosis [59,60]. Breast cancer cells become more aggressive and malignant with the loss of keratin as these proteins are replaced with vimentin, the intermediate filaments-protein of mesenchymal cells [61-63]. Experiments by Buhler demonstrated that highly invasive MDA-MB-231 breast cancer cells became less invasive and lacked tumorigenicity in nude mice with overexpression of keratin 18 [64]. It is, therefore, possible that DHA or DHA + CCM treatment may have transformed DMBA-induced tumors toward a more differentiated, less aggressive subtype. Furthermore, immune histological analysis of tumor tissues indicates that the DMBA-induced tumors were ER-negative and Her-2 positive, further validating the reported observations. We observed that DHA + CCM treatment caused a significant expression of ER in DMBA-induced tumors, further validating our observation of microarray data in SK-BR-3 cells (Table 3). Reversal of the estrogen negative to the estrogen positive phenotype has previously been described [65]. This observation also suggests that the combined treatment has induced differentiation in breast tumors. We have not been able to further characterize keratin or ER levels in these tumors due to scarcity of the tissue; however, these observations also need further investigation.

**Figure 9:**
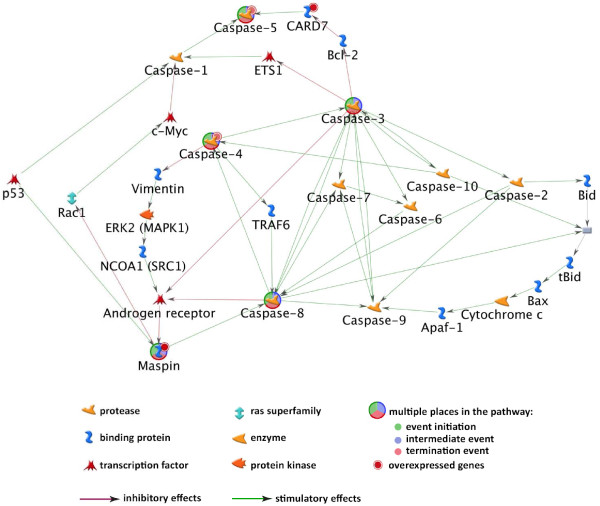
**Analysis of microarray data from SK-BR-3 cell line for interaction of maspin up- and down-stream mediators.** The microarray data from SK-BR-3 cell treatment with DHA, CCM, and DHA + CCM was used to determine the interaction of maspin with up- and down-regulatory mediators using MetaCoreTM version 6.3 (GeneGo). The interactions indicate maspin is regulated upstream by p53, whereas maspin is linked to activation of a number of caspase activities downstream for its prominent role in the induction of apoptosis.

One of the observations from our microarray data was the approximately 20-fold upregulation of *SERPINB5* and almost 60% downregulation of *BIRC5* genes. *SERPINB5* produces maspin, a tumor suppressor protein present in high concentrations in normal mammary epithelium and myoepithelium cells; maspin expression is reduced in primary breast cancers and is completely absent in invasive and metastatic tumor cells [66,67]. Data shown in Figure 8 indicate that maspin was absent or expressed at low levels in the tumors of control or DHA-fed animals. CCM treatment caused reexpression of maspin, and this expression appears to be further enhanced by the combined DHA + CCM diet. Reexpression of maspin in response to curcumin has previously been shown in breast cancer cells by Parsad et al. [68]. Maspin is a key regulatory molecule for the normal mammary gland and embryonic development [69]. The expression of *SERPINB5* is regulated at the transcriptional level through elements in the maspin promoter, particularly by p53 [70-72]. Maspin is present in the cytoplasm, but it translocates to the mitochondria and inhibits tumor progression through the mitochondrial apoptosis pathway [73]. Analysis of the microarray data for caspase-mediated downstream processes in SK-BR-3 cells, as shown in Figure 9, indicates that maspin expression was linked to the activation of a number of caspases involved in apoptosis. Additionally, maspin has also been shown to induce cell differentiation, which further contributes to its anti-cancer effects [74,75]. Furthermore, PPARγ induced mammary cell differentiation, which is also accompanied by enhanced maspin expression [76]; however, it is not known if PPARγ directly regulates maspin expression in cancer cells.

BIRC5 produces survivin, the smallest member (16 kDa) of the inhibitor of the apoptosis protein (IAP) family, which acts not only to inhibit apoptosis but also to control cell cycle progression [77-79]. Survivin is largely expressed in developing embryos and proliferating hematopoietic, epithelial, and gonadal cells [80]. It is mostly absent from well differentiated normal adult tissues, but hyperplasic regions of normal tissues often show some expression; however, survivin overexpression has been reported in nearly all human cancers, including breast cancer [80-82]. Data presented in Figure 8 indicate that DMBA-induced tumors expressed substantial levels of survivin. These levels were not affected by DHA or CCM treatment, but a combined treatment (DHA + CCM) caused almost a 50% reduction in survivin expression. Disrupting survivin expression or function in cancer cells has been shown to decrease cell proliferation by enhancing apoptosis. Survivin has been considered an effective target for anticancer strategies in several preclinical and early-phase clinical trials [83].

Factors that are involved in regulating maspin re-expression are also involved in regulating survivin expression. For example, nuclear factor kappaB (NFkB) upregulates survivin expression [84], whereas p53 and retinoblastoma protein (pRb) are required to repress survivin transcription [85]. More recently, Verhagen et al. reported that mutations of the p53 gene in breast carcinoma significantly correlate with an enhanced expression of survivin [86]. In addition, PPARγ reduces levels of survivin in different cancer types, including breast cancer [87,88].

**Figure 10:**
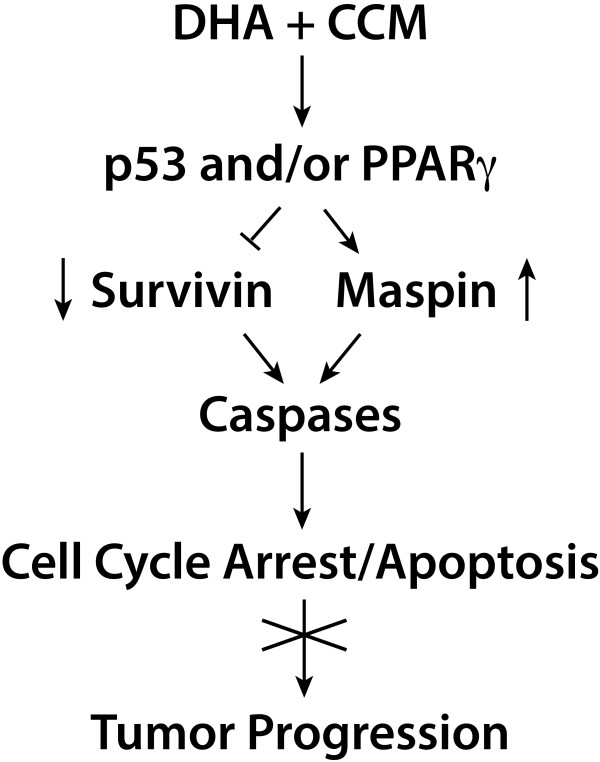
**The proposed signaling pathways for the synergistic effects of DHA and CCM on progression.** One possible mechanism for the effects of CCM, DHA or CCM + DHA on p53 activation and/or PPARγ expression is to suppress the anti-apoptotic protein, survivin, while increasing the expression of maspin, a tumor-suppressor protein. This situation could lead to the induction of apoptosis, thereby inhibiting tumor progression.

Previously, we demonstrated that DHA and CCM synergistically cause activation of p53 and upregulation of PPARγ expression. Based on these observations, it is possible that the effects of CCM + DHA on p53 activation and/or PPARγ expression cause suppression of the anti-apoptotic protein, survivin, with increased expression of maspin, a tumor suppressor protein. This effect would lead to the inhibition of cell cycle progression and to the induction of apoptosis, thereby inhibiting tumor progression (Figure 10). Clearly, additional experiments are needed to confirm a role of p53 and/or PPARγ on maspin re-expression and survivin suppression.

One limitation of this study is the low levels of linoleic acid in DHA and DHA + CCM diets. High levels of linoleic acid have been shown to stimulate breast cancer [89]. It is unlikely that low levels of linoleic acid have any effect on the growth or number of breast tumors since the DHA diet itself was not very effective. However, it is possible that reduced linoleic acid with CCM may have played a role in the synergistic effect of the DHA + CMM diet on breast tumor formation. Clearly, further investigation is required to determine the combined effect of a reduced level of linoleic and CCM on breast cancer growth.

## Conclusion

The data from this *in vitro* study is consistent with our previously published study. The results of this study further demonstrated that the synergistic effects of DHA + CCM were evident both under *in vitro* and *in vivo* conditions. SK-BR-3 cells and DMBA-induced tumors, both with ER^-^ and Her-2^+^ characteristics, were synergistically affected by DHA and CCM, which suggests that the specific breast cancer phenotype is an important factor for predicting efficacy. One possible mechanism for the synergistic effects of DHA + CCM on ER-/Her-2+ breast tumors involves the re-expression of maspin and the suppression of survivin.

## Abbreviations

BIRC5: Baculoviral inhibitor of apoptosis repeat-containing 5; CCM: Curcumin; CYP450: Cytochrome P450; DHA: Docosahexaenoic acid; DHASCO: Docosahexaenoic acid single cell oil; DMBA: 7,12-Dimethylbenz(a) anthracene; ER: Estrogen receptor; ESR-1: Estrogen receptor α gene; Her-2: Human epidermal, growth factor receptor 2; ERBB2: Her-2 gene; PR: Progresterone receptor; PGR: Progresterone receptor gene; SERPINB5: Serpin peptidase inhibitor, clade B (ovalbumin), member 5.

## Competing interests

The authors declare that they have no competing interests.

## Authors’ contributions

RAS, planned the study, supervised experiments, analyzed data and wrote manuscript; KH, participated in designing the experiments and analyzed tumor tissue samples; CW, performed *in vivo* study for tumor induction; JA, performed experiments for microarray analysis and designed *in vivo* experiments; ZX, analyzed diets and tissue samples on GC; CT, performed statistical analysis of data; IG, helped in establishing DMBA-induced *in vivo* model; YJ, performed histological analysis; CM, participated in designing the experiments, analyzing, evaluating and discussing data, and preparation of manuscript. All authors read and approved the final mauscript.

## Pre-publication history

The pre-publication history for this paper can be accessed here:

http://www.biomedcentral.com/1471-2407/13/418/prepub
